# Frontostriatal Circuit Dynamics Correlate with Cocaine Cue-Evoked Behavioral Arousal during Early Abstinence^1^,^2^,^3^

**DOI:** 10.1523/ENEURO.0105-16.2016

**Published:** 2016-06-23

**Authors:** Wesley C. Smith, Matthew H. Rosenberg, Leslie D. Claar, Victoria Chang, Sagar N. Shah, Wendy M. Walwyn, Christopher J. Evans, Sotiris C. Masmanidis

**Affiliations:** 1 Interdepartmental PhD Program in Neuroscience, University of California, Los Angeles, Los Angeles, California 90095; 2 Department of Neurobiology, University of California, Los Angeles, Los Angeles, California 90095; 3 Department of Bioengineering, University of California, Los Angeles, Los Angeles, California 90095; 4 Undergraduate Interdepartmental Program for Neuroscience, University of California, Los Angeles, Los Angeles, California 90095; 5 Department of Psychiatry and Biobehavioral Sciences, University of California, Los Angeles, Los Angeles, California 90095; 6 Brain Research Institute, University of California, Los Angeles, Los Angeles, California 90095; 7 Hatos Center for Neuropharmacology, Semel Institute for Neuroscience and Human Behavior, University of California, Los Angeles, Los Angeles, California 90095; 8 California Nanosystems Institute, University of California, Los Angeles, Los Angeles, California 90095

**Keywords:** cocaine, conditioned arousal, large-scale recordings, prefrontal cortex, pupillometry, striatum

## Abstract

It is thought that frontostriatal circuits play an important role in mediating conditioned behavioral responses to environmental stimuli that were previously encountered during drug administration. However, the neural correlates of conditioned responses to drug-associated cues are not well understood at the level of large populations of simultaneously recorded neurons, or at the level of local field potential (LFP) synchrony in the frontostriatal network. Here we introduce a behavioral assay of conditioned arousal to cocaine cues involving pupillometry in awake head-restrained mice. After just 24 h of drug abstinence, brief exposures to olfactory stimuli previously paired with cocaine injections led to a transient dilation of the pupil, which was greater than the dilation effect to neutral cues. In contrast, there was no cue-selective change in locomotion, as measured by the rotation of a circular treadmill. The behavioral assay was combined with simultaneous recordings from dozens of electrophysiologically identified units in the medial prefrontal cortex (mPFC) and ventral striatum (VS). We found significant relationships between cocaine cue-evoked pupil dilation and the proportion of inhibited principal cells in the mPFC and VS. Additionally, LFP coherence analysis revealed a significant correlation between pupillary response and synchrony in the 25–45 Hz frequency band. Together, these results show that pupil dilation is sensitive to drug-associated cues during acute stages of abstinence, and that individual animal differences in this behavioral arousal response can be explained by two complementary measures of frontostriatal network activity.

## Abstract (precis)

It is thought that frontostriatal circuits play an important role in mediating conditioned behavioral responses to environmental stimuli that were previously encountered during drug administration. However, the neural correlates of conditioned responses to drug-associated cues are not well understood at the level of large populations of simultaneously recorded neurons, or at the level of local field potential (LFP) synchrony in the frontostriatal network.

## Visual Overview

**Figure:**
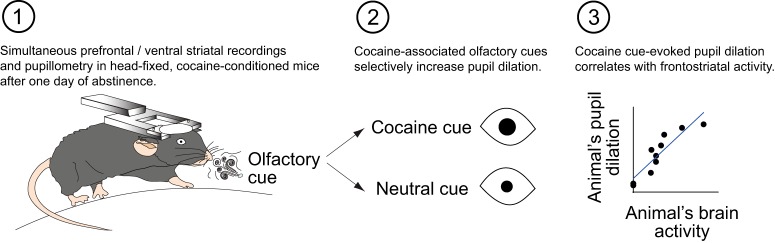

## Significance Statement

Frontostriatal circuits are implicated in drug craving and relapse, which can be triggered during abstinence by drug-associated cues. This study used large-scale neural recordings with silicon microprobe technology to simultaneously examine prefrontal and striatal activity in cocaine-conditioned mice. We found relationships among the level of cocaine cue-evoked neural inhibition, frontostriatal network synchrony, and conditioned arousal measured via pupillometry. This work shows that pupil dilation is a highly sensitive measure of arousal to cocaine cues. Furthermore, the results demonstrate the effectiveness of using large-scale recordings spanning multiple brain areas to examine the neural correlates of interindividual variability among drug-experienced animals.

## Introduction

Environmental stimuli formerly associated with drugs can have a powerful influence on behavior, presenting a long-term risk of relapse after the cessation of drug use (Robinson and Berridge, 1993; O'Brien et al., 1998). This is thought to occur because of functional changes in a variety of brain circuits mediating reward learning, motivation, arousal, and inhibitory behavioral control, which persist in drug abstinence (Everitt and Robbins, 2005; Britt et al., 2012; Muñoz-Cuevas et al., 2013; Lüthi and Lüscher, 2014; MacAskill et al., 2014; Pascoli et al., 2014). The medial prefrontal cortex (mPFC) and ventral striatum (VS) are two interconnected areas, which are believed to play a critical role in these processes (Jentsch and Taylor, 1999; Kalivas et al., 2005; Day and Carelli, 2007; Goldstein and Volkow, 2011). Neuroimaging studies in addicted human subjects have revealed that prefrontal and striatal regions are modulated by cocaine-associated cues (Maas et al., 1998; Childress et al., 1999; Wexler et al., 2001), and that some of these activation patterns correlate with self-reported drug craving (Bonson et al., 2002). Neuroimaging has also revealed alterations in frontostriatal network connectivity following drug use (Wilcox et al., 2011; Hu et al., 2015). However, because of the limited spatiotemporal resolution of neuroimaging techniques, little is known about the neurophysiological correlates of cocaine cue-evoked frontostriatal interactions. At the level of single-neuron measurements, units in the mPFC and VS are known to encode drug-associated cues (Carelli et al., 1993; Chang et al., 2000; Ghitza et al., 2003; Rebec and Sun, 2005; West et al., 2014). However, while individual animal differences in behavioral responses to drugs and their associated stimuli can be significant (Groman et al., 2012; Chen et al., 2013; Muñoz-Cuevas et al., 2013; Robinson et al., 2014; Storey et al., 2016), the neural correlates of this interindividual variability are not well understood. Large-scale recordings have the potential to identify novel relationships between neural activity in prefrontal and striatal circuits and drug cue-evoked behavior, but until now, such measurements have not been widely used together with animal models of addiction.

In addition to eliciting voluntary behavioral responses or subjectively measured craving, drug-associated cues are known to influence autonomic responses, indicating a heightened state of arousal in the form of heart rate and skin conductance changes (Ehrman et al., 1992) and pupil dilation (Rosse et al., 1995). Since the dynamics of prefrontal and other cortical circuits are related to pupil diameter (Aston-Jones and Cohen, 2005; Reimer et al., 2014; McGinley et al., 2015; Vinck et al., 2015; Joshi et al., 2016), which in turn reflects arousal (Hess and Polt, 1960; Bradley et al., 2008), we hypothesized that, during abstinence, drug-associated stimuli cause pupil dilation, that the degree of this effect varies among animals, and that this variability correlates with the dynamics of frontostriatal circuits.

To address this hypothesis, here we simultaneously performed large-scale *in vivo* neural recordings in the mPFC and VS together with a novel assay of conditioned arousal to cocaine-associated cues in mice in the early stage (first 24 h) of abstinence, which relies on pupil dilation measurements. We found that cocaine-paired olfactory cues induced a stronger pupillary response than saline-paired cues, and that this autonomic response was more selective for cocaine cues than the cue-evoked locomotion response. Large-scale recordings were performed with a customized 512 electrode silicon microprobe (Shobe et al., 2015), providing simultaneous measurements from dozens of electrophysiologically identified neurons in the mPFC and VS, as well as local field potential (LFP) oscillations. We assessed spontaneous and cue-evoked electrophysiological firing properties, and related the level of activation and inhibition to simultaneously measured changes in pupil diameter. We found that the proportion of prefrontal pyramidal cells and striatal projection neurons that were inhibited by cocaine-associated cues was correlated with the degree of pupil dilation. Moreover, we found that 25-45 Hz frontostriatal LFP coherence was correlated with the pupillary response. Together, these results suggest that suppression of frontostriatal activity, and elevated interregional synchrony, have a related role in triggering arousal to cocaine cues. These measurements provide new insights into the dynamics of frontostriatal circuits when drug-free animals are exposed to stimuli that were previously encountered during drug administration.

## Materials and Methods

### Animals and surgical procedures

All procedures were approved by the University of California, Los Angeles, Animal Care Committee. We used singly housed male C57BL/6J mice (12–16 weeks old; The Jackson Laboratory), with 10 mice receiving alternating cocaine and saline injections (referred to as the drug group) and 7 mice receiving only saline (referred to as the control group). Animals underwent an initial surgery under isoflurane anesthesia in a stereotactic apparatus to implant stainless steel head restraint bars bilaterally into their skulls using dental cement (Shobe et al., 2015). Animals were anesthetized with isoflurane for a second surgery on the evening prior to the day of the recording session to make a craniotomy over the anterior striatum and mPFC in the right hemisphere. The dura mater was opened to facilitate the insertion of microprobes during the recording session. An additional craniotomy was made over the posterior cerebellum for placement of a silver/silver chloride electrical reference wire. The craniotomies were covered with silicone elastomer sealant (Kwik-Cast, WPI) to seal the exposed skull.

### Cocaine conditioning

One week after the first surgery, animals began habituation to head restraint and the behavioral conditioning/testing room for 4 d, with one 30 min session per day. Animals were mounted with the head bar bracket on top of a 200-mm-diameter treadmill ball that was free to rotate forward and backward. No other stimuli were presented during habituation, except for low background lighting in the enclosed room provided by four 200 lumen LED lamps directed away from the eyes of the mice and toward the walls of the room. The lamps were necessary to prevent a saturation effect in the pupil dilation, which would occur in the absence of any light. After habituation, fully awake head-restrained animals were conditioned over 10 d to injections of cocaine or saline, paired with a previously unfamiliar olfactory cue. Each daily conditioning session for the drug group was devoted to either cocaine or saline, but never both on the same day. The drug group received alternating cocaine (15 mg/kg, 100 μl, s.c.) or saline (100 μl, s.c.) infusions administered over a 1 min period. The infusions were given by means of a syringe pump connected to a 27.5 gauge syringe needle inserted subcutaneously. We removed the needle after each session. To ensure consistency across training and behavioral testing (see next section), we also inserted the needle during the testing session, but it was disconnected from any fluid delivery system. Conditioning started with saline on day 1 and ended with cocaine on day 10. All cocaine or saline administration sessions were paired with a single continuous presentation of odorized air, starting 5 min preinjection and ending 25 min postinjection, when the animal was removed from the conditioning room and returned to its home cage in a vivarium housing facility. Olfactory cues were introduced via an olfactometer by bubbling air (0.15 L/min) through aromatic liquids (citral or isoamyl acetate) diluted 1:10 in mineral oil (Sigma-Aldrich), and mixing this product with a 1.5 L/min stream of air. This mixture flowed through a tube passing 15 mm away from, and perpendicularly to the animal’s nose, with a 5 mm opening in the tube aimed at the nose. Half of the drug group animals were counterbalanced and received the opposite drug–odor pairing. The saline group received saline injections during all 10 d of conditioning, paired with alternating citral and isoamyl acetate cues. During each conditioning session, we monitored behavior in the form of treadmill rotation velocity and pupil dilation. Pupillometry video was captured under infrared illumination with the camera (model acA640, Basler) focused on the right eye of the animal.

### Behavioral testing

The craniotomy surgery was performed 7–8 h after the final conditioning session. Behavioral testing and electrophysiological recordings were performed simultaneously in drug-free conditions, in animals abstinent from their last injection of cocaine (drug group) or saline (control group) for 24 ± 3 h. We provided animals a 15 min resting period without any cues, which was used to capture spontaneous activity. Subsequently, we randomly presented the previously paired odors (15 s in length, 30 trials per odor type, with a randomized mean intertrial interval of 20 ± 5 s) while monitoring treadmill locomotion and pupil dilation to assess behavioral responses.

### Electrophysiological recordings

A silicon microprobe (Shobe et al., 2015) was assembled targeting the medial prefrontal cortex and ventral striatum (256 electrodes per area, total of 512 electrodes). Each area was targeted with four silicon prongs spaced 0.2 mm apart, each containing 64 electrodes that were arranged in a hexagonal array pattern spanning 1.05 mm along the dorsal–ventral axis. Electrodes had an area of 100 μm^2^ and were gold plated (noncyanide gold solution, Sifco) to an impedance of 0.1–0.5 MΩ. In the mPFC, the relatively long span of the electrode array allowed us to simultaneously record from the prelimbic and infralimbic subregions. In the VS, the primary target was the nucleus accumbens core, but, due to the length of the electrode array, we also recorded from the region above the accumbens core (medial striatum). One to two hours prior to recording, the silicon microprobes were coated with a drop of fluorescent dye (DiD, Invitrogen) to assist with histological confirmation of their position. Animals were then mounted on the head restraint system. An electrical reference wire was placed on the cerebellar surface, covered in ACSF-saturated water-absorbing foam (Gelfoam), to improve electrical contact, and sealed with silicone elastomer (Kwik-Cast, WPI). The silicon microprobes were slowly inserted to stereotactically defined coordinates with a motorized micromanipulator (MP-285, Sutter Instruments). The tip position of the most medial shaft in the mPFC was targeted to 1.90 mm anterior, 0.07 mm lateral, and 3.40 mm ventral relative to bregma in the right hemisphere. The tip of the most medial shaft in the VS was placed in the same hemisphere at 1.0 mm anterior, 0.77 mm lateral, and 4.70 mm ventral relative to bregma. The insertion was monitored with a surgical microscope. After reaching the target depth, a drop of mineral oil was placed on the exposed cortical surface, and a 45 min settling period elapsed before beginning the electrophysiological data acquisition and behavioral testing (see the previous section). We monitored electrophysiological data at a sampling rate of 25 kHz/electrode, together with treadmill ball rotation and olfactory cue delivery time at a sampling rate of 10 kHz. Pupillometry video was synchronously captured at 25 frames/s. Following each recording, the microprobe was cleaned in trypsin solution (Invitrogen), rinsed with deionized water and ethanol, and reused throughout these experiments.

### Histology

Coronal brain sections were sliced at 100 μm on a vibratome, and individual sections were placed in order onto a 24 well plate containing ice-cold PBS solution. We then performed immunohistochemistry using standard procedures on the mPFC and striatal sections to determine the placement of the silicon prongs in each region. Sections were stained for neuronal nuclei with chicken-α NeuN primary antibodies (D3S3I, Cell Signaling Technology) and fluorescent α-chicken secondary antibodies (Alexa Fluor 488, Jackson ImmunoResearch). DiD, which diffusively labeled tissue near the probe insertion sites, was used to estimate the final probe position.

### Unit classification

Spike sorting was performed using a semi-automated spike waveform template-matching algorithm (Shobe et al., 2015). Each unit was assigned an estimated 3D coordinate as well as a histologically determined brain region. The estimated position coincided with the recording site exhibiting the highest spike amplitude for that unit. Finally, units were classified as principal neurons or interneurons in their respective brain regions. Striatal units with a minimum baseline rate of 0.02 Hz were classified as putative medium spiny projection neurons (MSNs), fast-spiking interneurons (FSIs), or tonically active neurons (TANs), based on spike waveform peak-to-trough width and the coefficient of variation of the baseline firing rate (Barthó et al., 2004; Bakhurin et al., 2016). FSIs were characterized by a narrow spike waveform (maximum width, 0.475 ms). MSNs and TANs both have wider waveforms (minimum width, 0.55 ms; maximum width, 1.25 ms). TANs were separated from MSNs by the regularity of their baseline firing (maximum coefficient of variation, 1.5). Units in the mPFC were classified as putative FSIs or pyramidal neurons using the same spike width separation criterion as described above (Tseng et al., 2006).

### Behavioral data analysis

Pupil diameter data were extracted from video frames with the MATLAB image analysis toolbox and custom scripts. To quantify behavioral responses during cocaine or saline conditioning, we calculated the mean postinjection treadmill rotation velocity and pupil diameter per animal. To quantify the cue-evoked behavioral responses during abstinence, for each animal we calculated the change in speed and pupil diameter during the 15 s cue presentation time relative to a 1 s baseline period prior to cue onset. Unless otherwise noted, responses were averaged across all 30 cocaine or saline-paired trials.

### Electrophysiological data analysis

The single-unit firing rate was calculated in time steps of 50 ms and smoothed by convolving with a Gaussian kernel (SD = 250 ms). Spontaneous firing activity was obtained by averaging the rate from the first 15 min of the recording, when animals received no olfactory cues and did not move on the treadmill. Cue-evoked activity was obtained by averaging across all cocaine- or saline-paired trials. To determine whether units were excited or inhibited by cues, we performed a permutation test to identify time bins whose firing rate was significantly different from a 1 s baseline period preceding the cue onset (criterion for significance, *p* < 0.01). If two or more consecutive time bins during the cue presentation period (*t* = 0–15 s from cue onset) were significantly greater (less) than the baseline, the unit was classified as excited (inhibited). Tables 1 and 2 list the number of total, excited, and inhibited pyramidal cells and MSNs per animal in the drug-treated group (*n* = 10 mice). Partitioning of units into different subregions was done using an objective criterion: all units recorded from the top half of the electrode array were assigned to one partition, and the remaining units were assigned to the other partition. We confirmed histologically that the top and bottom partitions in the mPFC corresponded approximately to the prelimbic and infralimbic cortices, respectively. In the VS, the top and bottom partitions corresponded approximately to the medial striatum and nucleus accumbens core, respectively. Local field potential signals were downsampled off-line to 1 kHz. To quantify the cue-evoked change in LFP coherence, for each animal we calculated the change in 25-45 Hz coherence during the 15 s cue presentation time relative to a 1 s baseline period prior to cue onset. Coherence changes were averaged across all cocaine- or saline-paired trials.

**Table 1:** Number of cortical pyramidal units per animal in the drug-treated group

Mouse ID	Total Pyramidal cells	Pyramidal cells excited by cocaine cues	Pyramidal cells inhibited by cocaine cues	Pyramidal cells excited by saline cues	Pyramidal cells inhibited by saline cues
1	84	18	26	11	23
2	141	13	21	14	28
3	87	17	15	16	10
4	57	3	6	14	24
5	58	16	7	5	5
6	95	22	15	8	5
7	31	6	10	1	6
8	67	10	6	11	10
9	27	4	1	0	2
10	39	8	5	12	0

**Table 2:** Number of striatal MSN units per animal in the drug-treated group

Mouse ID	Total MSNs	MSNs excited by cocaine cues	MSNs inhibited by cocaine cues	MSNs excited by saline cues	MSNs inhibited by saline cues
1	95	11	27	8	29
2	63	3	12	4	21
3	87	8	15	3	17
4	60	5	8	8	11
5	65	9	9	5	9
6	100	11	22	6	4
7	21	5	6	3	0
8	81	3	17	5	16
9	57	6	3	2	6
10	81	7	14	10	9

### Statistics

Table 3 contains a list of the statistical tests performed and their results. Permutation tests to determine significant cue-evoked excitation or inhibition of neural activity were performed with custom Matlab scripts (Shobe et al., 2015). All other statistical analysis was performed with standard Matlab functions or GraphPad Prism software. ANOVA was followed by Sidak’s correction for multiple comparisons. Analysis of neural activity in different electrode partitions was followed by Bonferroni’s correction for two comparisons, corresponding to the top and bottom partitions (α was adjusted to 0.025). Correlations between pupillary response and neural activity or synchrony were reported with both Pearson’s and Spearman’s correlation coefficients (*r* and *r*_s_, respectively) to ensure consistency. In all cases, Pearson’s and Spearman’s correlations were in agreement with regard to the statistical significance of the result (both types of correlations are shown in Table 3).

**Table 3:** Statistical table

	Figure	Data structure	Type of test	Sample size	*p* Value
a	Fig. 1D	Two factors (day and treatment)	Two-way ANOVA with repeated measures	*n* = 10 mice	Day: *F*_(4,72)_ = 3.752, *p* = 0.0079Treatment: *F*_(1,18)_ = 31.50, *p* < 0.0001Interaction: *F*_(4,72)_ = 2.639, *p* = 0.0407Sidak's multiple comparisons test:Saline vs drug, day 1: *p* = 0.2737Saline vs drug, day 2: *p* = 0.0630Saline vs drug, day 3: *p* = 0.0003Saline vs drug, day 4: *p* < 0.0001Saline vs drug, day 5: *p* < 0.0001
b	Fig. 1E	Two factors (day and treatment)	Two-way ANOVA with repeated measures	*n* = 10 mice	Day: *F*_(4,72)_ = 0.8313, *p* = 0.5096Treatment: *F*_(1,18)_ = 17.66, *p* = 0.0005Interaction: *F*_(4,72)_ = 0.6524, *p* = 0.6270Sidak's multiple comparisons test:Saline vs drug, day 1: *p* = 0.0148Saline vs drug, day 2: *p* = 0.0190Saline vs drug, day 3: *p* = 0.0004Saline vs drug, day 4: *p* = 0.0013Saline vs drug, day 5: *p* = 0.0034
c	Fig. 1F	Nonparametric	Wilcoxon matched pairs signed-rank test	*n* = 10 mice	*p* > 0.9999
d	Fig. 1G	Nonparametric	Wilcoxon matched pairs signed-rank test	*n* = 10 mice	*p* = 0.0273
e	Fig. 1H	Two variables	*r* and *r*_s_	*n* = 10 mice	*r* = 0.080, *p* = 0.827*r*_s_ = −0.103, *p* = 0.785
f	Fig. 1K	Nonparametric	Wilcoxon matched pairs signed-rank test	*n* = 10 mice	*p* = 0.6953
g	Fig. 1L	Nonparametric	Wilcoxon matched pairs signed-rank test	*n* = 10 mice	*p* = 0.9219
h	Fig. 2A	Two factors (day and treatment)	Two-way ANOVA with repeated measures	*n* = 7 mice	Day: *F*_(4,48)_ = 1.450, *p* = 0.2320Treatment: *F*_(1,12)_ = 0.02449, *p* = 0.8782Interaction: *F*_(4,48)_ = 0.4431, *p* = 0.7768Sidak's multiple comparisons test:Day 1: *p* > 0.9999Day 2: *p* = 0.9701Day 3: *p* > 0.9999Day 4: *p* > 0.9999Day 5: *p* = 0.8377
i	Fig. 2B	Two factors (day and treatment)	Two-way ANOVA with repeated measures	*n* = 7 mice	Day: *F*_(4,48)_ = 1.777, *p* = 0.1489Treatment: *F*_(1,12)_ = 0.07157, *p* = 0.7936Interaction: *F*_(4,48)_ = 0.2804, *p* = 0.8893Sidak's multiple comparisons test:Day 1: *p* = 0.9998Day 2: *p* = 0.9913Day 3: *p* = 0.9992Day 4: *p* > 0.9999Day 5: *p* = 0.9907
j	Fig. 2C	Nonparametric	Wilcoxon matched pairs signed-rank test	*n* = 7 mice	*p* = 0.2969
k	Fig. 2D	Nonparametric	Wilcoxon matched pairs signed-rank test	*n* = 7 mice	*p* = 0.3750
l	Fig. 4C, left	Nonparametric	Mann–Whitney test	*n* = 7 control group + 10 drug group	*p* = 0.4028Bonferroni’s correction: 2 comparisons,α = 0.025
m	Fig. 4C, right	Nonparametric	Mann–Whitney test	*n* = 7 control group + 10 drug group	*p* = 0.0328Bonferroni’s correction: 2 comparisons,α = 0.025
n	Fig. 4D, left	Nonparametric	Mann–Whitney test	*n* = 7 control group + 10 drug group	*p* = 0.5942Bonferroni’s correction: 2 comparisons,α = 0.025
o	Fig. 4D, right	Nonparametric	Mann–Whitney test	*n* = 7 control group + 10 drug group	*p* = 0.4705Bonferroni’s correction: 2 comparisons,α = 0.025
p	Fig. 4E, left	Nonparametric	Mann–Whitney test	*n* = 7 control group + 10 drug group	*p* = 0.9252Bonferroni’s correction: 2 comparisons,α = 0.025
q	Fig. 4E, right	Nonparametric	Mann–Whitney test	*n* = 7 control group + 10 drug group	*p* = 0.4028Bonferroni’s correction: 2 comparisons,α = 0.025
r	Fig. 4F, left	Nonparametric	Mann–Whitney test	*n* = 7 control group + 10 drug group	*p* = 0.3130Bonferroni’s correction: 2 comparisons,α = 0.025
s	Fig. 4F, right	Nonparametric	Mann–Whitney test	*n* = 7 control group + 10 drug group	*p* > 0.9999Bonferroni’s correction: 2 comparisons,α = 0.025
t	Fig. 4G, left	Nonparametric	Kolmogorov–Smirnov test	*n* = 26 control + 80 drug group FSIs	*p* = 0.7Bonferroni’s correction: 2 comparisons,α = 0.025

u	Fig. 4G, right	Nonparametric	Kolmogorov–Smirnov test	*n* = 21 control + 60 drug group FSIs	*p* = 0.945Bonferroni’s correction: 2 comparisons,α = 0.025
v	Fig. 4H, left	Nonparametric	Kolmogorov–Smirnov test	*n* = 102 control + 110 drug group FSIs	*p* = 0.0584Bonferroni’s correction: 2 comparisons,α = 0.025
w	Fig. 4H, right	Nonparametric	Kolmogorov–Smirnov test	*n* = 42 control + 97 drug group FSIs	*p* = 0.349Bonferroni’s correction: 2 comparisons,α = 0.025
x	Fig. 5E	Nonparametric	Wilcoxon matched-pairs signed rank test	*n* = 10 mice	*p* = 0.4316
y	Fig. 5F	Nonparametric	Wilcoxon matched-pairs signed rank test	*n* = 10 mice	*p* = 0.6953
z	Fig. 5G	Nonparametric	Wilcoxon matched-pairs signed rank test	*n* = 10 mice	*p* = 0.1602
aa	Fig. 5H	Nonparametric	Wilcoxon matched-pairs signed rank test	*n* = 10 mice	*p* = 0.9102
ab	Fig. 6A	Two variables	*r* and *r*_s_	*n* = 10 mice	*r* = 0.2132, *p* = 0.5543*r*_s_ =0.1394, *p* = 0.7072
ac	Fig. 6B	Two variables	*r* and *r*_s_	*n* = 10 mice	*r* = 0.3706, *p* = 0.2918*r*_s_ = 0.3091, *p* = 0.3869
ad	Fig. 6C	Two variables	*r* and *r*_s_	*n* = 10 mice	*r* = 0.8089, *p* = 0.0046*r*_s_ = 0.7818, *p* = 0.0105
ae	Fig. 6D	Two variables	*r* and *r*_s_	*n* = 10 mice	*r* = 0.7793, *p* = 0.0079*r*_s_ = 0.7091, *p* = 0.0268
af	Fig. 6E	Two variables	*r* and *r*_s_	*n* = 10 mice	*r* = 0.9051, *p* = 0.0003*r*_s_ = 0.9605, *p* < 0.0001Bonferroni’s correction: 2 comparisons,α = 0.025
ag	Fig. 6F	Two variables	*r* and *r*_s_	*n* = 10 mice	*r* = 0.7426, *p* = 0.0139*r*_s_ = 0.7455, *p* = 0.0174Bonferroni’s correction: 2 comparisons,α = 0.025
ah	Fig. 6G	Two variables	*r* and *r*_s_	*n* = 10 mice	*r* = 0.5701, *p* = 0.0853*r*_s_ = 0.5152, *p* = 0.1334Bonferroni’s correction: 2 comparisons,α = 0.025
ai	Fig. 6H	Two variables	*r* and *r*_s_	*n* = 10 mice	*r* = 0.8391, *p* = 0.0024*r*_s_ = 0.7818, *p* = 0.0105Bonferroni’s correction: 2 comparisons,α = 0.025
aj	Fig. 7A	Two variables	*r* and *r*_s_	*n* = 10 mice	*r* = 0.1131, *p* = 0.7558*r*_s_ = −0.0901, *p* = 0.8113
ak	Fig. 7B	Two variables	*r* and *r*_s_	*n* = 10 mice	*r* = 0.1634, *p* = 0.6520*r*_s_ = 0.2727, *p* = 0.4483
al	Fig. 7C	Two variables	*r* and *r*_s_	*n* = 10 mice	*r* = −0.1255, *p* = 0.7297*r*_s_ = −0.0909, *p* = 0.8113
am	Fig. 7D	Two variables	*r* and *r*_s_	*n* = 10 mice	*r* = −0.3601, *p* = 0.3068*r*_s_ = −0.3455, *p* = 0.3304
an	Fig. 7E	Two variables	*r* and *r*_s_	*n* = 10 mice	*r* = −0.1352, *p* = 0.7096*r*_s_ = 0.006061, *p* > 0.9999
ao	Fig. 7F	Two variables	*r* and *r*_s_	*n* = 10 mice	*r* = 0.3674, *p* = 0.2963*r*_s_ = 0.4061, *p* = 0.2475
ap	Fig. 7G	Two variables	*r* and *r*_s_	*n* = 10 mice	*r* = 0.05994, *p* = 0.8694*r*_s_ = 0.1394, *p* = 0.7072
aq	Fig. 7H	Two variables	*r* and *r*_s_	*n* = 10 mice	*r* = 0.2834, *p* = 0.4275*r*_s_ = 0.2485, *p* = 0.4918
ar	Fig. 8C	Two variables	*r* and *r*_s_	*n* = 10 mice	*r* = 0.8011, *p* = 0.0053*r*_s_ = 0.8909, *p* = 0.0011
as	Fig. 8D	Two variables	*r* and *r*_s_	*n* = 10 mice	*r* = 0.4686, *p* = 0.1719*r*_s_ = 0.5879, *p* = 0.0806

## Results

### Conditioned pupil dilation by cocaine-associated cues

**Figure 1.**
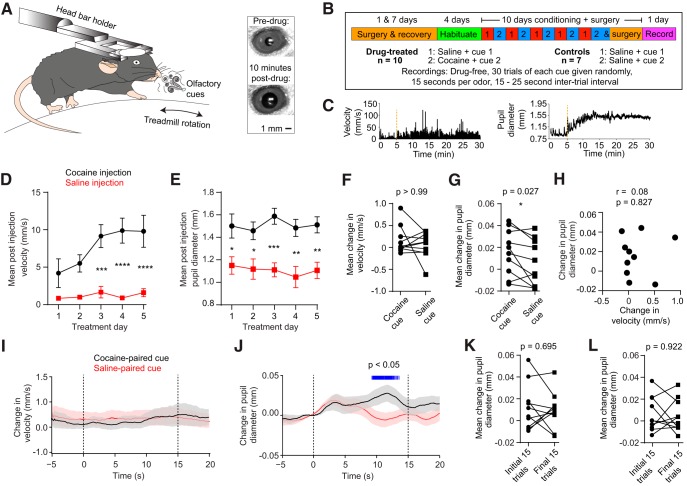
Enhancement of cocaine cue-evoked pupil dilation during drug abstinence. ***A***, Left, Setup for drug conditioning using olfactory cues in head-restrained mice. Behavior was monitored in the form of pupil dilation and circular treadmill rotation velocity. Right, Image of the pupil of an animal before drug injection (top) or 10 min after a single injection of cocaine during conditioning (bottom). ***B***, Timeline of surgery, habituation, drug conditioning, and recording. ***C***, Time dependence of absolute value of treadmill rotation velocity (left) and pupil diameter (right) for one representative animal on the first day of cocaine conditioning. Dashed line indicates the time of drug injection. ***D***, For the drug group (*n* = 10), postinjection treadmill rotation velocity showed a significant effect of treatment type (two-way ANOVA, *F*_(1,18)_ = 31.50, *p* < 0.0001) and day of treatment (*F*_(4,72)_ = 3.75, *p* = 0.008). ***E***, Postinjection pupil diameter showed a significant effect of treatment type (two-way ANOVA, *F*_(1,18)_ = 17.66, *p* = 0.0005), while there was no effect of day of treatment (*F*_(4,72)_ = 0.831, *p* = 0.510). ***F***, When presented with the cues on the test day in the absence of cocaine, drug-experienced mice (*n* = 10) showed an equal locomotor response to cocaine- and saline-associated cues (Wilcoxon matched-pairs signed rank test, *p* > 0.99). ***G***, Drug-experienced mice showed a higher pupil dilation in response to cocaine-associated cues (Wilcoxon matched-pairs signed rank test, *p* = 0.027). ***H***, Cocaine cue-mediated pupil dilation changes were not correlated with treadmill rotation changes (*r* = 0.08, *p* = 0.827). ***I***, Cue-triggered treadmill rotation velocity vs time averaged across drug-treated mice shows no difference in locomotion between cocaine- and saline-paired cues. Dashed lines indicate cue onset and offset. ***J***, Mean cue-triggered change in pupil dilation vs time. Blue lines indicate time bins where the cocaine and saline cues elicited significantly different dilation (paired *t* test, *p* < 0.05). ***K***, There was no significant difference in pupillary response between the initial 15 trials and final 15 trials of the cocaine cue (Wilcoxon matched pairs signed-rank test, *p* = 0.695). ***L***, There was no significant difference in pupillary response between the initial 15 trials and the final 15 trials of the saline cue (Wilcoxon matched pairs signed-rank test, *p* = 0.922). Data in ***D***, ***E***, ***I***, and ***J*** are reported as the mean ± SEM. *p < 0.05, **p < 0.01, ***p < 0.001, ****p < 0.0001.

We developed a behavioral assay of arousal in response to cocaine-associated cues in fully awake, head-restrained mice (Fig. 1*A*). Before recording, mice in the drug group (*n* = 10) were conditioned over a 10 d period with alternating injections of cocaine and saline, which were paired with a specific olfactory cue (Fig. 1*B*). During this period, mice showed a psychomotor sensitization effect, as marked by increased rotational movement on the treadmill after doses of cocaine (Fig. 1*C*, left, *D*; two-way ANOVA: effect of treatment type: *F*_(1,18)_ = 31.50, *p* < 0.0001; effect of treatment day: *F*_(4,72)_ = 3.75, *p* = 0.008; interaction effect: *F*_(4,72)_ = 2.64, *p* = 0.041). At the same time, mice showed increased pupil dilation in response to cocaine injections, which did not significantly change over the course of administration (Fig. 1*C*, right, *E*; two-way ANOVA: effect of treatment type: *F*_(1,18)_ = 17.66, *p* = 0.0005; effect of treatment day: *F*_(4,72)_ = 0.831, *p* = 0.510; interaction effect: *F*_(4,72)_ = 0.65, *p* = 0.627).

After conditioning, animals were prepared for concomitant electrophysiological recording and behavioral testing. During behavioral testing, 24 h drug-abstinent animals were randomly exposed to olfactory cues previously associated with either cocaine or saline, but were not given any injections. While some animals moved in response to cues, we found no difference in cue-evoked treadmill rotation velocity between cocaine- and saline-paired odors (Fig. 1*F*; Wilcoxon matched pairs signed-rank test, *p* > 0.99). In contrast, we found a significantly higher change in pupil diameter following the presentation of cocaine-paired cues (Fig. 1*G*; Wilcoxon matched pairs signed-rank test, *p* = 0.027). There was no correlation between cue-evoked changes in pupil dilation and locomotion in response to the cocaine-associated odor (Fig. 1*H*, *r* = 0.08, *p* = 0.827). Figure 1, *I* and *J*, shows the time dependence of average velocity and pupil diameter, respectively. Finally, we compared the mean pupillary response of the initial 15 trials and the final 15 trials, and found no consistent change in response to the drug cue (Fig. 1*K*; Wilcoxon matched pairs signed-rank test, *p* = 0.695) or saline cue (Fig. 1*L*; Wilcoxon matched pairs signed-rank test, *p* = 0.922), suggesting that elevated arousal in response to drug-associated cues is maintained throughout behavioral testing.

**Figure 2.**
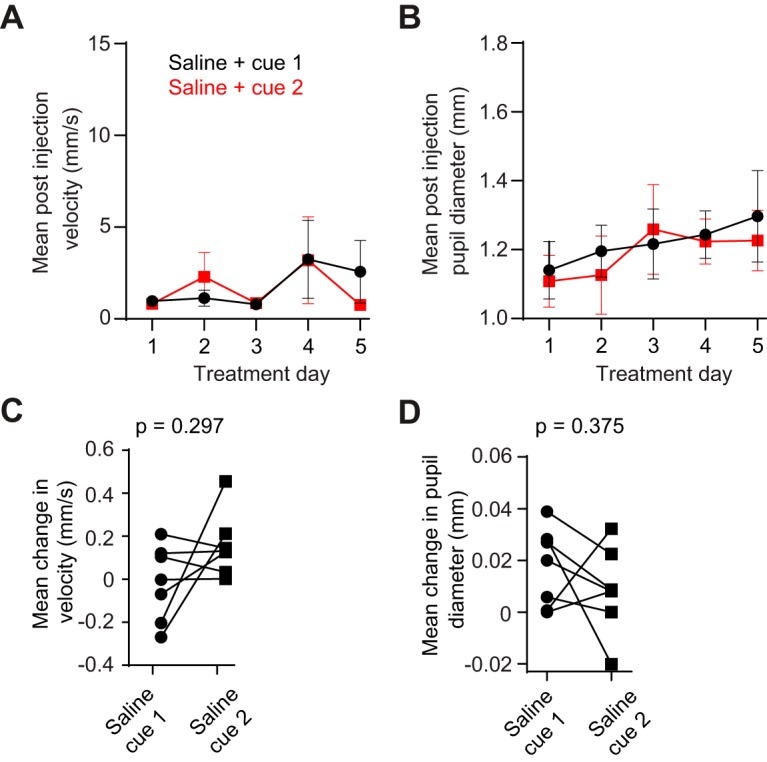
Control mice do not show differences in cue-evoked pupil dilation. ***A***, For the control group (*n* = 7), postinjection treadmill rotation velocity showed no effect of odor type (two-way ANOVA, *F*_(1,12)_ = 0.024, *p* = 0.878) or day of treatment (*F*_(4,48)_ = 1.450, *p* = 0.232). ***B***, Control group animals showed no effect of odor type (*F*_(1,12)_ = 0.072, *p* = 0.794) or day of treatment (*F*_(4,48)_ = 1.777, *p* = 0.149). ***C***, When presented with cues on the test day, control group mice (*n* = 7) showed an equal locomotor response to both cues (Wilcoxon matched-pairs signed rank test, *p* = 0.297). ***D***, When presented with cues on the test day, control group mice showed an equal pupillary response to both cues (Wilcoxon matched-pairs signed rank test, *p* = 0.375). Data in ***A*** and ***B*** are reported as the mean ± SEM.

We confirmed our results by repeating conditioning and testing with a group of control animals (*n* = 7) that only received saline injections paired with the same two odors (Fig. 1*B*). Control animals showed no change in locomotion as a function of either odor type or day of treatment (Fig. 2*A*; two-way ANOVA, *p* > 0.05 for all effects). Similarly, no changes were observed in pupil diameter during conditioning (Fig. 2*B*; two-way ANOVA, *p* > 0.05 for all effects). On the test day, we found no statistical difference in either cue-evoked locomotion (Fig. 2*C*; Wilcoxon matched pairs signed-rank test, *p* = 0.297) or pupil diameter (Fig. 2*D*; Wilcoxon matched pairs signed-rank test, *p* = 0.375). These results show that during short-term abstinence, cocaine-associated olfactory cues selectively increase behavioral arousal in the form of pupil dilation, but do not selectively influence locomotion.

### Cocaine does not alter average spontaneous and cue-evoked neural dynamics early in abstinence

**Figure 3.**
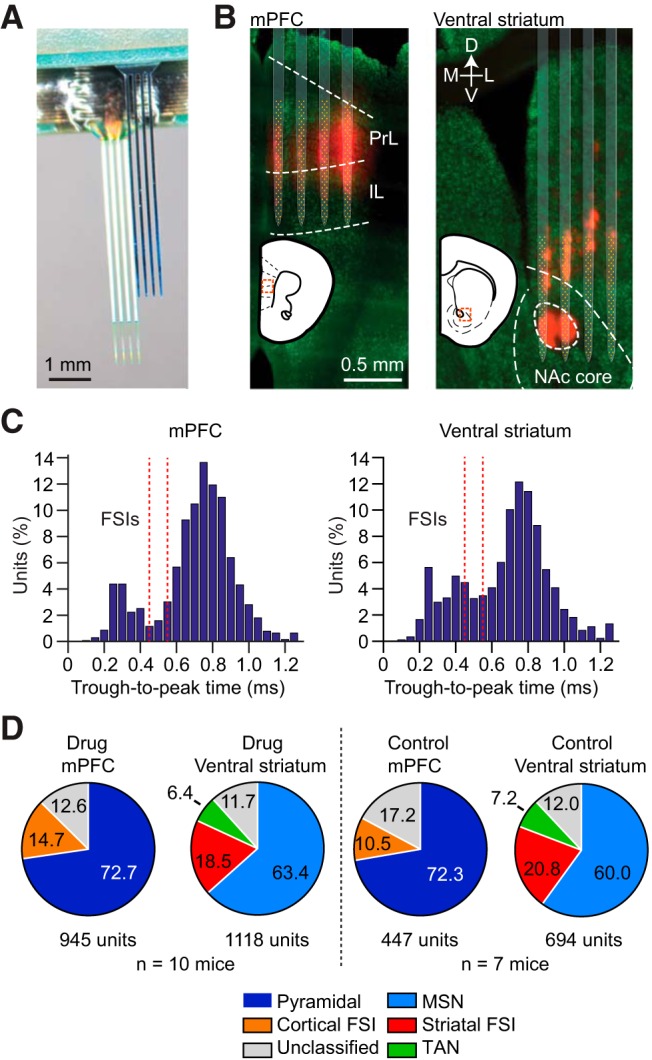
Large-scale neural recordings in the frontostriatal circuit. ***A***, A 512 electrode silicon microprobe targeting the mPFC and VS. ***B***, Representative reconstruction of electrode placement in the mPFC (left) and VS (right) from the same animal. Red, DiD dye; green, NeuN staining. ***C***, Distribution of trough-to-peak spike duration in the mPFC and VS. Red dashed lines denote the classification boundary between FSI and non-FSI units (units between the lines are unclassified). ***D***, Proportion and total number of cells recorded in the mPFC and VS in the drug and control groups.

To monitor frontostriatal network dynamics in abstinent drug-conditioned animals, we developed a 512 electrode silicon microprobe enabling large-scale simultaneous recordings in the mPFC and VS (Fig. 3*A*). Correct electrode placement was confirmed histologically (Fig. 3*B*). Units in each area were classified as principal cells (pyramidal cells in the mPFC, and MSNs in the VS) or interneurons based on spike waveform width (see Materials and Methods; Fig. 3*C*). We recorded a total of 2063 single units from drug-treated mice and 1141 units from control mice. In drug-treated mice, 72.7% of mPFC neurons fit our criteria for being pyramidal neurons, 14.7% were putative FSIs, and 12.6% were unclassified. In drug-treated mice, 63.4% of VS neurons fit our criteria for MSNs, 18.5% were putative FSIs, 6.4% were putative TANs, and 11.7% were unclassified (Fig 3*D*). In control mice, 72.3% of mPFC neurons fit our criteria for being pyramidal neurons, 10.5% were putative FSIs, and 17.2% were unclassified. In control mice, 60% of VS neurons fit our criteria for MSNs, 20.8% were putative FSIs, 7.2% were putative TANs, and 12% were unclassified.

**Figure 4.**
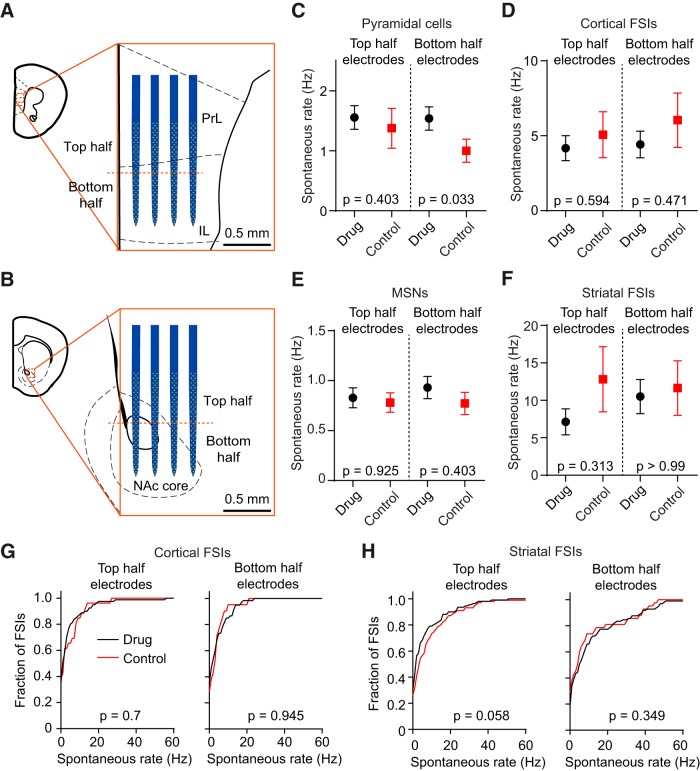
Cocaine does not alter spontaneous cortical or striatal firing rate in early abstinence. ***A***, ***B***, Illustration of how units in the mPFC and VS were partitioned according to their location on the top or bottom half of the electrode array. ***C*–*F***, There was no difference between the average firing rate of principal cells or FSIs in any of the subregions examined (Mann–Whitney *U* test, *p* > 0.025; exact probability values are listed in the figure, α was adjusted to 0.025 after Bonferroni’s correction for two comparisons, corresponding to the top and bottom partitions). Data in ***C*–*F*** are reported as the mean ± SEM firing rate across the animals in the drug (*n* = 10) and control (*n* = 7) groups. ***G***, ***H***, There was no difference in the pooled spontaneous FSI firing rate in any of the subregions examined (Kolmogorov–Smirnov test, *p* > 0.05; exact probability values are listed in the figure).

Cocaine exposure is frequently associated with prefrontal hypoactivity (Trantham et al., 2002; Chen et al., 2013). Furthermore, the prelimbic and infralimbic regions of the mPFC are often associated with opposing roles in cocaine-seeking behavior (Peters et al., 2008). To examine spontaneous firing properties in these subregions, we selected units from the top and bottom halves of the electrode array (Fig. 4*A*, cortex, *B*, striatum), and compared the mean spontaneous firing rate per animal of different neuronal populations between the drug and control groups. After correcting for two comparisons, we did not find a significant difference in the spontaneous firing rate of cocaine-treated animals in any of the cortical and striatal subregions examined (Fig. 4*C–F*; Mann–Whitney *U* test, *p* > 0.025, none of the results are significant after adjusting α to 0.025 following Bonferroni’s multiple comparisons correction). When comparing the mean spontaneous rate per animal, the relatively small number (*n* = 3–5) of FSIs recorded in certain subregions of some animals may have contributed to the large variability of our FSI results, making it challenging to detect an effect of cocaine. Therefore, we also performed a comparison after pooling all FSIs in the drug and control groups. Again, we found no significant difference in firing rate between these groups (Fig. 4*G*,*H*; Kolmogorov–Smirnov test, *p* > 0.05). These results demonstrate that for the areas targeted with our electrodes, cocaine does not significantly alter the spontaneous cortical or striatal activity of principal neurons and FSIs early in abstinence.

**Figure 5.**
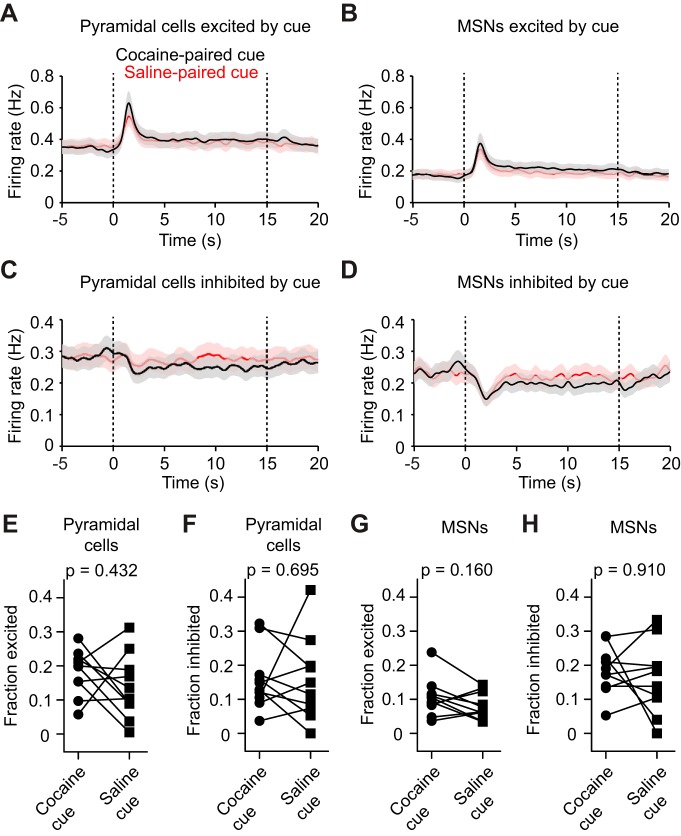
Average cue-evoked activity is not selective for cocaine-associated stimuli. ***A***, ***B***, Mean cue-triggered firing rate of all significantly excited pyramidal cells and MSNs, combined across data from the drug treatment group. Black and red lines, respectively, indicate the response to cocaine- and saline-paired cues. Dashed lines indicate cue onset and offset. There was no significant difference between the response to cocaine- and saline-paired cues at any time point (paired permutation test, *p* > 0.05). ***C***, ***D***, Mean cue-triggered firing rate of all significantly inhibited pyramidal cells and MSNs, combined across data from the drug treatment group. There was no significant difference between the response to cocaine- and saline-paired cues at any time point (paired permutation test, *p* > 0.01). Data in ***A–D*** are reported as the mean ± SEM. ***E***, ***F***, There was no difference in the average fraction of cortical pyramidal cells excited or inhibited by cocaine cues (Wilcoxon matched pairs signed-rank test, *p* > 0.05). ***G***, ***H***, There was no difference in the average fraction of MSNs excited or inhibited by cocaine cues (Wilcoxon matched pairs signed-rank test, *p* > 0.05). Data in ***E*–*H*** denote the fraction of cells per animal in the drug group (*n* = 10).

We next examined cue-evoked neural activity. A portion of principal neurons in the mPFC and VS showed significant excitation or inhibition in response to cocaine-associated olfactory stimuli (Fig. 5*A–D*), demonstrating that neural activity is modulated by olfactory cues. However, there was no difference in the fraction of neurons that was excited or inhibited by cocaine versus saline cues (Fig. 5*E–H*; all *p* > 0.05, paired Wilcoxon signed-rank test). These results show that, on average, these circuits do not appear to preferentially encode cocaine-associated cues.

### Conditioned pupillary response correlates with frontostriatal inhibition

**Figure 6.**
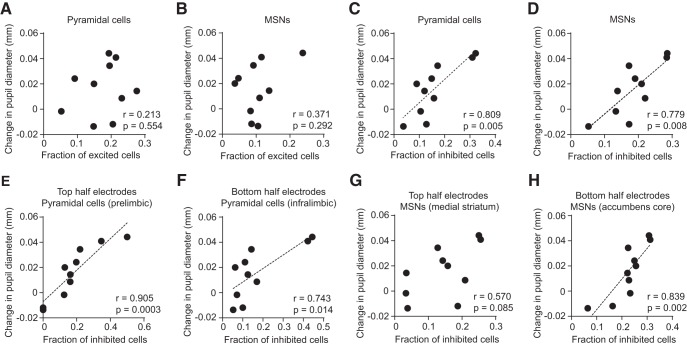
Cocaine cue-evoked pupil dilation correlates with frontostriatal inhibition. ***A***, There was no correlation of mean change in pupillary response to the fraction of cortical pyramidal neurons excited by the cocaine-paired cue (*r* = 0.213, *p* = 0.554). ***B***, There was no correlation of mean change in pupillary response to the fraction of MSNs excited by the cocaine-paired cue (*r* = 0.371, *p* = 0.292). ***C***, The mean change in pupillary response was correlated with the fraction of inhibited pyramidal neurons (*r* = 0.809, *p* = 0.005). ***D***, The mean change in pupillary response was correlated with the fraction of inhibited MSNs (*r* = 0.779, *p* = 0.008). Results from ***A*–*D*** were obtained by combining cortical or striatal principal cells across the entire electrode array. ***E***, ***F***, Inhibited units in both the prelimbic and infralimbic cortex maintained their relationship to pupillary response (Pearson’s correlation, *p* < 0.025; α was adjusted to 0.025 after Bonferroni’s correction for two comparisons; exact probability values are listed in the figure). ***G***, Inhibited MSNs in the medial striatum were uncorrelated to pupillary response (*r* = 0.570, *p* = 0.085). ***H***, Inhibited MSNs in the nucleus accumbens core were correlated with pupillary response (*r* = 0.839, *p* = 0.002). Plots show data from the drug group (*n* = 10).

Since we did not find differences in the mean level of cocaine- and saline-associated cue encoding in the mPFC and VS, we next tested whether the variability in behavioral responses among individual drug-experienced animals (*n* = 10) could account for the observed patterns of neural activity. Since drug-paired cues selectively affected pupillary responses, but not treadmill locomotion, we focused on individual animal changes in pupil diameter in response to cocaine cue presentation. We found that the mean change in pupil diameter was unrelated to the fraction of significantly excited pyramidal neurons (Fig. 6*A*, *r* = 0.213, *p* = 0.554) and MSNs (Fig. 6*B*, *r* = 0.371, *p* = 0.292). In contrast, there was a significant positive correlation between pupil diameter change and the fraction of inhibited pyramidal neurons (Fig. 6*C*, *r* = 0.809, *p* = 0.005) and MSNs (Fig. 6*D*, *r* = 0.779, *p* = 0.008). These results were obtained by combining units from all electrode depths in the mPFC and VS. To examine whether these relationships are consistent within different cortical and striatal subregions, in each area we partitioned units into two groups according to their location on the electrode, as previously described (see Materials and Methods; Fig. 4*A*,*B*). In the mPFC, the fraction of inhibited units in both the top and bottom halves of the electrode array, corresponding approximately to the prelimbic and infralimbic cortex, maintained a relationship between the fraction of inhibited cells and the change in pupil diameter (Fig. 6*E*, *r* = 0.905, *p* = 0.0003; Fig. 6*F*, *r* = 0.743, *p* = 0.014). In the VS, the fraction of units recorded from the top half of electrodes (corresponding to the medial striatum) showed no correlation to behavior (Fig. 6*G*, *r* = 0.571, *p* = 0.085), whereas the fraction of units from the bottom half (corresponding to the nucleus accumbens core) inhibited by the drug cue remained highly correlated to pupillary response (Fig. 6*H*, *r* = 0.839, *p* = 0.002). These results suggest that during early drug abstinence, inhibition of the medial prefrontal cortex and the nucleus accumbens core is involved in conditioned arousal to drug-associated cues. In contrast, medial striatal MSNs do not appear to have such a relationship with behavior.

**Figure 7.**
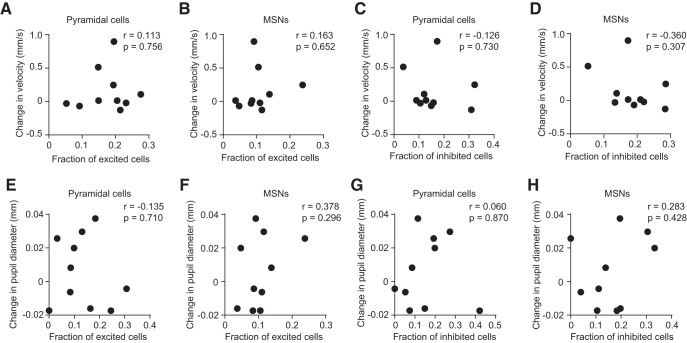
No correlation of neural activity with cocaine cue-evoked treadmill speed or saline cue-evoked pupil dilation. ***A***, There was no correlation of the mean change in treadmill velocity with the fraction of cortical pyramidal neurons excited by the cocaine-paired cue (*r* = 0.113, *p* = 0.756). ***B***, There was no correlation of the mean change in treadmill velocity with the fraction of MSNs excited by the cocaine-paired cue (*r* = 0.163, *p* = 0.652). ***C***, There was no correlation of the mean change in treadmill velocity with the fraction of cortical pyramidal neurons inhibited by the cocaine-paired cue (*r* = −0.126, *p* = 0.73). ***D***, There was no correlation of the mean change in treadmill velocity with the fraction of MSNs inhibited by the cocaine-paired cue (*r* = −0.36, *p* = 0.307). ***E***, There was no correlation of the mean change in pupillary response with the fraction of cortical pyramidal neurons excited by the saline-paired cue (*r* = −0.135, *p* = 0.701). ***F***, There was no correlation of the mean change in pupillary response with the fraction of MSNs excited by the saline-paired cue (*r* = 0.367, *p* = 0.296). ***G***, There was no correlation of the mean change in pupillary response with the fraction of cortical pyramidal neurons inhibited by the saline-paired cue (*r* = 0.060, *p* = 0.869). ***H***, There was no correlation of the mean change in pupillary response with the fraction of MSNs inhibited by the saline-paired cue (*r* = 0.283, *p* = 0.428). Results from ***A*–*H*** were obtained by combining cortical or striatal principal cells across the entire electrode array. Plots show data from the drug group (*n* = 10).

We next looked for correlations between the fraction of excited or inhibited cells, and treadmill running evoked by cocaine-paired cues. There was no significant correlation (Fig. 7*A–D*, *p* > 0.05; exact probability values are in the figure legend), suggesting that frontostriatal activity is more related to conditioned arousal in the form of pupil dilation than it is to conditioned locomotion.

To determine whether neural activity has any relationship with saline cue-evoked pupil dilation, we also looked for correlations between the fraction of excited or inhibited cells and pupil dilation in response to saline-paired cues. There was no statistically significant correlation in any of these comparisons (Fig. 7*E–H*, *p* > 0.05). Thus, while we found significant correlations between cocaine cue-evoked frontostriatal inhibition and pupil dilation, there was no corresponding relationship of saline cue-evoked activity with pupil dilation. This is consistent with brain circuit alterations following drug exposure that selectively mediate conditioned arousal responses to drug-associated stimuli, but not neutral stimuli.

### Conditioned pupillary response correlates with frontostriatal LFP coherence

**Figure 8.**
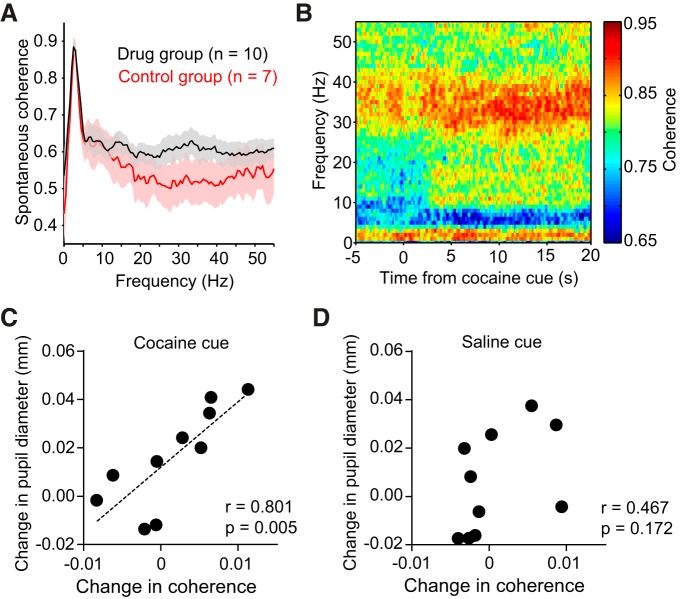
Cocaine cue-evoked pupil dilation correlates with 25–45 Hz frontostriatal LFP coherence. ***A***, Frontostriatal coherence spectra of spontaneous LFP activity. Black and red lines, respectively, indicate the mean ± SEM of spectra recorded from animals in the drug (*n* = 10) and control (*n* = 7) groups. Note the peak at ∼25–45 Hz for the cocaine group. ***B***, LFP coherence spectrogram from one animal showing modulation of coherence by olfactory stimuli. Data are aligned to cocaine cue onset. ***C***, The mean change in pupillary response was correlated with the change in 25–45 Hz LFP coherence during the presentation of cocaine cues (*r* = 0.801, *p* = 0.005). ***D***, The mean change in pupillary response was uncorrelated with the change in 25–45 Hz LFP coherence during the presentation of saline cues (*r* = 0.467, *p* = 0.172).

Since the mPFC projects to the VS (Berendse et al., 1992; Voorn et al., 2004), neural activity in these areas is related (Chang et al., 2000; Ishikawa et al., 2008). To gain insight into the significance of this interaction for our behavioral task, we examined whether neural synchrony in the form of LFP coherence was related to cue-evoked arousal measured via pupillary responses. LFP signals were measured together with single-unit action potentials on the same electrodes. Frontostriatal LFP coherence spectra revealed a peak in coupling between the mPFC and VS at ∼25-45 Hz both under resting conditions (Fig. 8*A*) and during cocaine cue presentations (Fig. 8*B*). These rhythms are within the frequency range of LFP signals that were previously found to be sensitive to cocaine administration (McCracken and Grace, 2013). Across the animals tested in the drug group (*n* = 10), we found a significant correlation between the mean change in cocaine cue-evoked 25–45 Hz coherence and pupil diameter (Fig. 8*C*, *r* = 0.801, *p* = 0.005). This relationship held even after partitioning LFP signals from the top and bottom half of the electrode array in each area (results not shown; *p* < 0.025, all correlations remain significant after adjusting α to 0.025 following Bonferroni’s correction for two comparisons). Finally, we did not find an analogous relationship between the change in saline cue-evoked LFP coherence and pupil diameter (Fig. 8*D*, *r* = 0.467, *p* = 0.172). As shown in Figure 7*E–H*, this is consistent with a conditioned arousal response to drug-associated stimuli, but not neutral stimuli.

## Discussion

This study introduced an assay of behavioral responses to cocaine-associated olfactory stimuli, which was specifically developed to support large-scale electrophysiological recordings in awake head-restrained mice. The assay used measurements of pupil dilation and treadmill movement. Pupillary response is an autonomic behavior that is modulated by the state of arousal of an animal (Hess and Polt, 1960; Bradley et al., 2008; Vinck et al., 2015). Pupil dilation is influenced by locomotion but can also be modulated independent of movement (Vinck et al., 2015), providing two related but separable approaches for tracking how mice respond to cocaine injection or cocaine-associated cues. Specifically, as drug conditioning progressed animals responded to cocaine injections with increased locomotion on a circular treadmill, consistent with a psychomotor sensitization effect (Robinson and Berridge, 2008). Pupil dilation was also impacted by cocaine administration, but this response did not change significantly as drug conditioning progressed, which may reflect a ceiling effect after just the first injection. After a 24 h drug abstinence period, there was greater pupil dilation in response to odors previously paired with cocaine than with saline, whereas there was no difference in locomotion on the treadmill. This demonstrates that under the conditions of our behavioral test, pupillary response is more sensitive to cocaine cues than locomotion, at least in the early stage of drug abstinence. The most parsimonious interpretation of these results is that animals are more aroused by odors previously paired with cocaine because of an associative learning process that occurred during the conditioning period (O'Brien et al., 1998). We therefore presume that interindividual variations in the amplitude of the pupil dilation effect reflect differences in conditioned arousal. An open question of this study is whether cue-evoked pupil dilation is related to elevated drug craving. Analogous effects have been shown in studies with addicted human subjects using other autonomic responses (heart rate and skin conductance; Ehrman et al., 1992). Thus, while we cannot be certain that the mice in this study craved drugs, the conditioned arousal response appears to be consistent with elevated craving.

To the best of our knowledge, our study is the first use of pupillometry to assess responses to drug-paired cues in mice. The rapid occurrence of the dilation effect (within 24 h after the last drug administration) presents a sensitive, noninvasive method to measure drug cue arousal in models of addiction. Furthermore, since pupillometry is compatible with neuroimaging methods in human subjects (Bray et al., 2008), the task introduced here has potentially useful applications in human addiction research and diagnosis.

We combined this behavioral assay with silicon microprobe technology to simultaneously record from dozens of electrophysiologically identified prefrontal and striatal units. We initially focused on differences in spontaneous neural activity between drug- and control-treated mice 24 h after the last cocaine injection. We found that spontaneous activity in the areas examined was unaltered. Cocaine use frequently has been associated with prefrontal hypoactivity (Jentsch and Taylor, 1999; Trantham et al., 2002; Sun and Rebec, 2006; Chen et al., 2013). From our data, we infer that hypoactivity is not a strong feature of the circuits we examined in the first 24 h of cocaine abstinence. In fact, we even noted a tentative trend toward higher pyramidal cell firing in the infralimbic portion of the PFC (Fig. 4*C*; Mann–Whitney *U* test, *p* = 0.033, which is not significant after adjusting α to 0.025 following Bonferroni’s correction for two comparisons), suggesting that short-term and long-term states of abstinence may be characterized by different states of resting brain activity. An additional consideration is that the electrophysiological recordings and spike sorting are inherently biased toward more spontaneously active neurons. Thus, it is possible that this analysis did not take into account relatively silent units.

We also examined cue-evoked neural activity, focusing on units with significant excitatory or inhibitory responses. Our results show that, on average, cocaine and saline cue-evoked frontostriatal network dynamics were not discernibly different in the early stage of drug abstinence. Previous studies suggest a somewhat complex activation pattern involving a mixture of enhanced and reduced activity in prefrontal and striatal networks by drug-associated cues (Ghitza et al., 2003; Goldstein and Volkow, 2011; Mahler and Aston-Jones, 2012; West et al., 2014). Several factors may contribute to our observed level of cue-modulated neural activity in the frontostriatal network. The first consideration is that the relatively short duration of abstinence in our study (24 h) does not induce significant incubation effects (Pickens et al., 2011). An increase in cue-evoked VS neural firing has been observed after extended (30 d) periods of abstinence (Hollander and Carelli, 2007; West et al., 2014). A second factor may be that the recording sites in the VS targeted regions that do not discriminately encode cocaine from neutral cues (Ghitza et al., 2003).

While neither the mPFC nor the VS selectively encoded cocaine cues on average, we noticed a substantial variability in the neural response between individual animals. We therefore tested whether these results could be explained by variations in the behavioral responses of individual animals. We found that changes in pupil diameter in response to cocaine-paired odors were significantly correlated with the overall fraction of inhibited principal cells in the mPFC (including both prelimbic and infralimbic areas) and the nucleus accumbens core. At the same time, we found that the fraction of excited units was not predictive of behavior. These results show that inactivation (or active suppression) of medial prefrontal and ventral striatal output is related to an elevated autonomic response to cocaine-paired stimuli.

Noradrenergic signaling from the locus ceruleus is strongly implicated in behavioral arousal (Aston-Jones and Cohen, 2005). The locus ceruleus is reciprocally connected with the cortex (Chandler et al., 2014; Schwarz et al., 2015), providing a possible route for mediating the observed relationship between pupil diameter and neural activity. The potential importance of these reciprocal interactions is supported by findings of prefrontal modulation of locus ceruleus activity (Sara and Hervé-Minvielle, 1995; Jodo et al., 1998), and electrophysiological studies showing relationships between cortical dynamics and pupil dilation (Reimer et al., 2014; McGinley et al., 2015; Vinck et al., 2015; Joshi et al., 2016). In addition to noradrenergic input, frontostriatal circuits are likely to be modulated by dopaminergic signaling (Everitt and Robbins, 2005; Goldstein and Volkow, 2011).

It is worth noting that neural inhibition in both the prelimbic and infralimbic aspects of the mPFC exhibited a significant relationship with pupil dilation. This suggests that the suppression of activity in these regions is linked to higher levels of arousal in response to cocaine cues. This finding may appear to be at odds with studies showing opposing roles for these regions in cocaine seeking, with the prelimbic and infralimbic cortex, respectively, driving and suppressing this behavior (Peters et al., 2008; LaLumiere et al., 2010). On the other hand, there is also evidence of a less dichotomous role of these regions in drug- and natural reward-related behavior (Moorman and Aston-Jones, 2015; Moorman et al., 2015). Furthermore, it is possible that, due to the considerable functional diversity of the prefrontal cortex (Fuster, 2001; Pessoa, 2008), this circuit differentially controls autonomic and voluntary responses to drug-associated cues.

The mPFC projects to the VS (Berendse et al., 1992), which in turn can indirectly influence cortical activity via mesocorticolimbic feedback loops (Everitt and Robbins, 2005). We observed coherent LFP oscillations between the mPFC and VS, which are thought to synchronize VS activity to afferent signals from the mPFC (McCracken and Grace, 2013). The strength of this coherence in the 25–45 Hz frequency band was found to be correlated with the degree of cocaine cue-evoked pupil dilation, suggesting that the observed neural dynamics in the mPFC and VS were related to frontostriatal network interactions.

Together, this study used large-scale neural recordings in mice to examine neurophysiological properties related to drug abstinence. The high throughput of these measurements combined with a novel behavioral assay of cocaine cue arousal, enabled us to identify new relationships between frontostriatal network dynamics and behavioral responses to cocaine-associated stimuli. Our finding that cue-evoked pupil dilation is related to lower activity in the mPFC and VS, as well as increased LFP coherence between these structures, may provide new insights for understanding and preventing drug relapse. These results also highlight the importance of accounting for interindividual behavioral variability in studies of addiction, which can be significant (Groman et al., 2012; Chen et al., 2013; Muñoz-Cuevas et al., 2013; Storey et al., 2016). Finally, the compatibility of our behavioral task with awake head-restrained mice creates new opportunities for studying systems-level neural dynamics in rodent models of addiction using a variety of complementary recording and brain circuit dissection tools.

## Synthesis

The decision was a result of the Reviewing Editor Christian Lüscher and the peer reviewers coming together and discussing their recommendations until a consensus was reached. A fact-based synthesis statement explaining their decision and outlining what is needed to prepare a revision is listed below. The following reviewers agreed to reveal their identity: Francois Georges, Vincent Pascoli.

In general the authors make a convincing case that pupil diameter can be used to monitor cocaine cues.

However the following points should be addressed:

1. The authors carried out large-scale recordings (more than 3000 cells) in identified sub-population of prefrontal cortex and nucleus accumbens, but draw they conclusion on a fraction of the recorded cells corresponding to cue-inhibited neurons in the prefrontal cortex and in the nucleus accumbens. The number of principal neurons showing a significant excitation or inhibition in response to cocaine-associated cues is hidden (or at least not clearly mentioned) through the manuscript . Authors should clarify this information and report the accurate number of inhibited (and excited) neurons per animal. This information is crucial to evaluate the robustness of the data.

2. An unexpected result is that a 10 days cocaine-conditioning period does not alter spontaneous activity of prefrontal and striatal neurons. Authors should consider that the stress of the second anesthesia for the craniotomy performed 24h before the recording session may affect the spontaneous activity of the prefrontal and straiatal neurons. A group of mice should be tested with the craniotomy performed during the conditioning period (i.e. a couple of day prior the recording session). The surgery for craniotomy should be mentioned in figure 1B.

3. Fast-spiking interneurons in the nucleus accumbens play a key role for the integration of cortical information and control of MSN neurons. Authors should consider increasing the number of striatal FSIs recorded in control condition to decrease the standard deviation and reavaluate the effect of cocaine abstinence on average spontaneous activity (Fig 4F).

4. In the introduction it doesn't clearly appear why the authors are interested in individual differences to drug cues. Authors might have to give examples of published data in which such observations were made. And, why they want study this variability between animals and why pupil diameter is a good candidate to reflect the variability in sensitivity to the drug-paired cues among drug-treated mice.

5.It looks like velocity response is also variable. Could authors make correlation with neural activity also for this behavioural parameter?

6. Fig4C :It looks that there is a tendency for an increased spontaneous activity in the IL in drug-treated animals that should be mentioned and discussed in the manuscript.

7. Authors only speak about the VS (Accumbens) in the manuscript while for the last figure reader realize that they in fact compare Accumbens Core to the ventral part of the Dorsal striatum, due to the placement of the recording electrode. Authors should give explanations for recordings in the Dorsal Striatum in the context of drugs. Why not comparing Core and shell instead. This would have been more logical given the connection from IL and PL. If not possible, the panels with subregions of the VS should be removed.
